# Multi-Scale Anisotropic Yield Function Based on Neural Network Model

**DOI:** 10.3390/ma18030714

**Published:** 2025-02-06

**Authors:** Hongchun Shang, Lanjie Niu, Zhongwang Tian, Chenyang Fan, Zhewei Zhang, Yanshan Lou

**Affiliations:** 1 Science and Technology on Electromechanical Dynamic Control Laboratory, Xi’an 710065, China; 2 Xi’an Institute of Electromechanical Information Technology, Xi’an 710065, China; 3 Military Representative Bureau of Army Equipment Department in Xi’an, Xi’an 710065, China; 4 School of Mechanical Engineering, Xi’an Jiaotong University, Xi’an 710065, China

**Keywords:** neural network, anisotropic yield function, multi-scale modeling, crystal plasticity, finite element analysis

## Abstract

The increasingly complex form of traditional anisotropic yield functions brings difficulties to parameter calibration and finite element application, and it is necessary to establish a unified paradigm model for engineering applications. In this study, four traditional models were used to calibrate the anisotropic behavior of a 2090-T3 aluminum alloy, and the corresponding yield surfaces in $\left(\sigma_{xx},\sigma_{yy},\sigma_{xy}\right)$ and $\left(\alpha ,\beta ,r\right)$ spaces were studied. Then, *α* and *β* are selected as input variables, and *r* is regarded as an output variable to improve the prediction and generalization capabilities of the fully connected neural network (FCNN) model. The prediction results of the FCNN model are finally compared to the calibration results of the traditional model, and the reliability of the FCNN model to predict the anisotropy is verified. Then, the data sets with different stress states and loading directions are generated through crystal plasticity finite element simulation, and the yield surface is directly predicted by the FCNN model. The results show that the FCNN model can accurately reflect the anisotropic characteristics. The anisotropic yield function based on the FCNN model can cover the characteristics of all traditional models in one subroutine, which greatly reduces the difficulty of subroutine development. Moreover, the finite element subroutine based on the FCNN model can model anisotropic behaviors.

## 1. Introduction

Accurate prediction of anisotropic plastic behavior is of a great significance for the reliability of numerical analysis in sheet metal forming processes. A variety of yield functions have been proposed to describe the anisotropic plastic deformation of metals in recent decades. The first anisotropic yield function was introduced by Hill [1], and many anisotropic yield functions have been proposed to simulate the anisotropic plastic behavior of metals. Barlat et al. [2] introduced a six-parameter fourth-order linear transformation tensor to expand the isotropic yield function to an anisotropic form, and this method has since been widely used in anisotropic yield functions [3,4]. Yoshida et al. [5] proposed a six-order polynomial 3D yield function based on the Drucker function, which accurately predicted the *r*-values and flow stress directionality. In addition, the applicability of non-associative flow rules in anisotropic characterization is confirmed, which further improves the accuracy of sheet metal forming simulation [6]. To describe the anisotropy and strength differential effect, Hu et al. [7] proposed a yield criterion for anisotropic-asymmetric materials based on normalized stress invariance. Du et al. [8] proposed an improved yield criterion to describe the strength differential effect and anisotropic hardening based on hydrostatic pressure. Hou et al. [9] constructed a plasticity model with non-correlated fourth-order polynomial functions, which captured the anisotropy of yield stress and plastic flow under the plane strain state. Soare [10] proposed an interpolation data-based model based on the data-driven plasticity framework to model the yield and flow characteristics of metals.

Artificial intelligence is an important driver of technological revolution and industrial transformation. Deep learning is an algorithm that learns and represents data sets based on artificial neural network (ANN) architecture [11]. In the field of metal plasticity, the latest progress in constitutive models is based on in-depth research on basic physics at the atomic level, mainly on dislocation dynamics [12,13] and grain evolution [14]. On the other hand, neural networks provide an alternative to data calibration for the development of phenomenological constitutive models, including hardening models [15,16], yield equations [17,18], fracture criteria [19,20], and forming limits [21].

The yield criterion can be classified into crystal plasticity and phenomenological constitutive models according to different construction theories. Compared to phenomenological models, crystal plasticity models are more complex and require longer calculation time. Thus, phenomenological constitutive models have become increasingly important in engineering applications. Fazily and Yoon [22] used a neural network to learn the stress integration process based on the data set numerically generated by the Euler inverse method, which is applicable to quadratic and non-quadratic anisotropic yield functions under plane stress. Zhang and Mohr [23] mathematically derived the relationship between strain and stress based on the traditional regression mapping scheme, so that the relationship between strain and stress can be modeled by a neural network function. Heidenreich et al. [18] combined micromechanics with homogenization theory to predict the effective mechanical properties of materials based on microstructural information.

Many studies have been conducted to relate plastic behavior to microstructure through ANN techniques. Due to the high dimensionality of the microstructure space and multi-objective design requirements, Liu et al. [24] inverted the relationship between microstructure and mechanical properties through an ANN to find the optimal structure. Muhammad et al. [25] revealed strain fields in tensile experiments of additively manufactured aluminum based on process parameters and microstructural information. A convolutional neural network was used by Ibragimova et al. [26] to predict local stress distributions and strain distributions in polycrystals. Yamanaka et al. [27] demonstrated a virtual data generation method for material modeling, and deep neural networks were used to estimate the biaxial stress–strain curves of aluminum alloys. Ali et al. [28,29] coupled the fully connected neural network (FCNN) model with the crystal plasticity finite element method (CPFEM) to predict the stress–strain behavior and texture evolution of a 6063-T6 aluminum alloy under uniaxial tension and pure shear. The robustness of the prediction model can be improved by introducing energy constraints, thereby stabilizing the prediction in the presence of incomplete or noisy data [30].

This research proposes a yield function framework based on a neural network model to characterize the anisotropic behavior of metals during plastic deformation. The shapes of the yield surfaces in $\left(\sigma_{xx},\sigma_{yy},\sigma_{xy}\right)$ space and $\left(\alpha ,\beta ,r\right)$ space are discussed, and the corresponding relationship between different stress states and anisotropy is pointed out. Higher-quality training data are obtained through the variable conversion to accurately construct the mapping relationship between *α*, *β*, and *r*, and the yield stress and *r*-values are calculated according to the prediction results of the FCNN model. The proposed FCNN framework is coupled with four macroscopic traditional yield functions to simulate the anisotropy of a 2090-T3 aluminum alloy (AA2090-T3). Based on the simulation results of microscopic CPFEM as the input data set, the FCNN model is directly trained to obtain the corresponding yield surface. A comprehensive discussion on further improvement of the current research is given at the end.

## 2. Application of the Yield Functions to AA2090-T3

### 2.1. The Mises Yield Equation in Two Spaces

Yield functions can be classified based on whether they study isotropic materials or anisotropic materials. The earliest yield functions like the Tresca yield function and von Mises yield function are about isotropic materials, but as the material and manufacturing technology develops, theses yield functions are unable to describe anisotropic materials, promoting the development of anisotropic yield functions. Hill proposed the famous Hill48 yield function, and most anisotropic yield functions are developed based on it. To describe the yield function more clearly, *x*, *y*, *z* are the rolling direction (RD), diagonal direction (DD), and transverse direction (TD), respectively. The stress state is presented in the following form:

(1) $$\sigma =\left[\begin{array}{ccc} \sigma_{xx} & \sigma_{xy} & \sigma_{xz}\\ \sigma_{yx} & \sigma_{yy} & \sigma_{yz}\\ \sigma_{zx} & \sigma_{zy} & \sigma_{zz} \end{array}\right]$$

Normalized stresses are chosen, so the three stress components in the Cartesian axis system (anisotropic axes *x*, *y*, *z* with *x* axis along the rolling direction) are

(2) $$\begin{array}{c} \sigma_{xx}^{*}=cos\beta cos\alpha \\ \sigma_{yy}^{*}=cos\beta sin\alpha \\ \sigma_{xy}^{*}=sin\beta \end{array}$$

The von Mises yield function under plane stress is simplified as follows:

(3) $$\sqrt{{\sigma_{xx}^{*}}^{2}+{\sigma_{yy}^{*}}^{2}-\sigma_{xx}^{*}\sigma_{yy}^{*}+3{\sigma_{xy}^{*}}^{2}}=C$$

**Figure 1:**
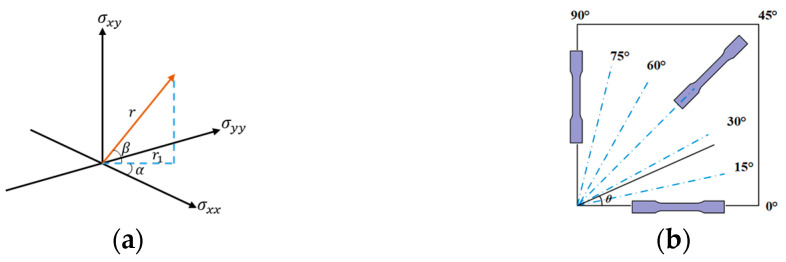
Schematic diagram of stress components and rolling direction: (**a**) relationship between $\sigma_{xx},\sigma_{yy},\sigma_{xy}$ stress components and α,β,r variables in stress space, (**b**) loading directions of experiments for the characterization of anisotropic plastic behaviors.

The material constant *C* is the yield stress measured by the simplest uniaxial tensile test. Therefore, the three stress components based on the Mises yield function are expressed as

(4) $$\begin{array}{c} \sigma_{xx}=\sigma_{xx}^{*}/C\\ \sigma_{yy}=\sigma_{yy}^{*}/C\\ \sigma_{xy}=\sigma_{xy}^{*}/C \end{array}$$

where the azimuth angle is α∈[0,2π], the polar angle is β∈[−π/2,π/2] and the radial coordinate is r≥0. According to the geometric relationship in Figure 1a, *r* is calculated according to Equation (5) as

(5) $$r=\sqrt{{\sigma_{xx}}^{2}+{\sigma_{yy}}^{2}+{\sigma_{xy}}^{2}}$$

More importantly, the following relationship exists between the $\sigma_{xx},\sigma_{yy},\sigma_{xy}$ stress components, and the α,β,r variables:

(6) $$\frac{\sigma_{xx}}{\sqrt{\sigma_{xx}^{2}+\sigma_{yy}^{2}}}=\cos\left(\alpha \right)$$

(7) $$\frac{\sqrt{\sigma_{xx}^{2}+\sigma_{yy}^{2}}}{r}=\cos\left(\beta \right)$$

Consider a uniaxial tensile test with dogbone specimens cut out of an anisotropic sheet metal with its axial direction *θ* $\left(\theta \in \left[0,\pi /2\right]\right)$ away from the rolling direction. The uniaxial tensile yield stress is denoted as $\sigma_{\theta }$. Then, the stress components in the material coordinate are computed by the stress transformation equation as

(8) $$\left\{\begin{array}{c} \sigma_{xx}=\sigma_{\theta }{cos}^{2}\theta \\ \sigma_{yy}=\sigma_{\theta }{sin}^{2}\theta \\ \sigma_{xy}=\sigma_{\theta }sin\theta cos\theta \end{array}\right.$$

An anisotropic yield function under plane stress is given in a general form of

(9) $$\overset{ˉ}{\sigma }=f\left(\sigma_{xx},\sigma_{yy},\sigma_{xy}\right)=\sigma_{\theta }f\left({cos}^{2}\theta ,{sin}^{2}\theta ,sin\theta cos\theta \right)$$

Then, the uniaxial tensile yield stress along *θ* away from RD, $\sigma_{\theta }$, is solved as

(10) $$\sigma_{\theta }=\frac{\overset{ˉ}{\sigma }}{f\left({cos}^{2}\theta ,{sin}^{2}\theta ,sin\theta cos\theta \right)}$$

For the uniaxial tensile test with *θ* away from RD, the longitudinal and width strain increments are $d\varepsilon_{\theta }^{p}$ and $d\varepsilon_{\theta +90}^{p}$, respectively. According to the assumption that the volume keeps constant during the plastic deformation, the thickness plastic strain is computed as

(11) $$d\varepsilon_{t}^{p}=-d\varepsilon_{\theta }^{p}-d\varepsilon_{\theta +90}^{p}=-d\varepsilon_{xx}^{p}-d\varepsilon_{yy}^{p}$$

According to the definition of the Lankford parameter, the *r*-value of the uniaxial tensile test along *θ* from RD is computed as

(12) $$r_{\theta }=\frac{d\varepsilon_{w}^{p}}{d\varepsilon_{t}^{p}}=-\frac{d\varepsilon_{\theta +90}^{p}}{d\varepsilon_{\theta }^{p}+d\varepsilon_{\theta +90}^{p}}=-\frac{d\varepsilon_{\theta +90}^{p}}{d\varepsilon_{xx}^{p}+d\varepsilon_{yy}^{p}}$$

According to the flow rule in Equation (13),

(13) $$d\varepsilon_{ij}^{p}=d\lambda \frac{\partial f\left(\sigma_{ij}\right)}{{\partial \sigma }_{ij}}$$

(14) $$\left\{\begin{array}{c} d\varepsilon_{\theta +90}^{p}=d\lambda \frac{\partial f}{{\partial \sigma }_{\theta +90}}\\ d\varepsilon_{xx}^{p}=d\lambda \frac{\partial f}{{\partial \sigma }_{xx}}\\ d\varepsilon_{yy}^{p}=d\lambda \frac{\partial f}{{\partial \sigma }_{yy}} \end{array}\right.$$

from Equation (8),

(15) $$\left\{\begin{array}{c} \frac{\partial \sigma_{xx}}{{\partial \sigma }_{\theta +90}}={cos}^{2}\left(\theta +90^{{}^{\circ}}\right)={sin}^{2}\theta \\ \frac{{\partial a}_{yy}}{{\partial \sigma }_{\theta +90}}={sin}^{2}\left(\theta +90^{{}^{\circ}}\right)={cos}^{2}\theta \\ \frac{{\partial \sigma }_{xy}}{{\partial \sigma }_{\theta +90}}=\sin\left(\theta +90^{{}^{\circ}}\right)\cos\left(\theta +90^{{}^{\circ}}\right)=-\sin\theta \cos\theta \end{array}\right.$$

by means of the chain rule of the differentiation, $\frac{\partial f}{{\partial \sigma }_{\theta +90}}$ is obtained as

(16) $$\begin{array}{c} \frac{\partial f}{{\partial \sigma }_{\theta +90}}=\frac{\partial f}{{\partial \sigma }_{xx}}\frac{{\partial \sigma }_{xx}}{{\partial \sigma }_{\theta +90}}+\frac{\partial f}{{\partial \sigma }_{yy}}\frac{{\partial \sigma }_{yy}}{{\partial \sigma }_{\theta +90}}+\frac{\partial f}{{\partial \sigma }_{xy}}\frac{{\partial \sigma }_{xy}}{{\partial \sigma }_{\theta +90}}\\ \;\;\;=\frac{\partial f}{{\partial \sigma }_{xx}}{sin}^{2}\theta +\frac{\partial f}{{\partial \sigma }_{yy}}{cos}^{2}\theta -\frac{\partial f}{{\partial \sigma }_{xy}}\sin\theta \cos\theta \end{array}$$

Accordingly, the *r*-value of the uniaxial tensile test along *θ* from RD, $r_{\theta }$, is finally obtained as

(17) $$r_{\theta }=-\frac{\frac{\partial f}{{\partial \sigma }_{xx}}{sin}^{2}\theta +\frac{\partial f}{{\partial \sigma }_{yy}}{cos}^{2}\theta -\frac{\partial f}{{\partial \sigma }_{xy}}sin\theta cos\theta }{\frac{\partial f}{{\partial \sigma }_{xx}}+\frac{\partial f}{{\partial \sigma }_{yy}}}$$

The stress components of the other stress states in the material coordinate are computed as follows:

Shear yield stress:

(18) $$\sigma_{xx}=\sigma_{SS_{\theta }}sin2\theta ,\sigma_{yy}=-\sigma_{SS_{\theta }}sin2\theta ,\sigma_{xy}=\sigma_{SS_{\theta }}cos2\theta$$

Plane strain tensile yield stress:

(19) $$\sigma_{xx}=\left(3+cos2\theta \right)\frac{\sigma_{PST_{\theta }}}{4},\sigma_{yy}=\left(3-cos2\theta \right)\frac{\sigma_{PST_{\theta }}}{4},\sigma_{xy}=-\frac{\sigma_{PST_{\theta }}}{4}sin2\theta$$

Uniaxial compressive yield stress:

(20) $$\sigma_{xx}=-\sigma_{UC_{\theta }}{cos}^{2}\theta ,\sigma_{yy}=-\sigma_{UC_{\theta }}{sin}^{2}\theta ,\sigma_{xy}=-\sigma_{UC_{\theta }}sin\theta cos\theta$$

Equibiaxial tension yield stress:

(21) $$\sigma_{xx}=\sigma_{EBT},\sigma_{yy}=\sigma_{EBT},\sigma_{xy}=0$$

**Figure 2:**
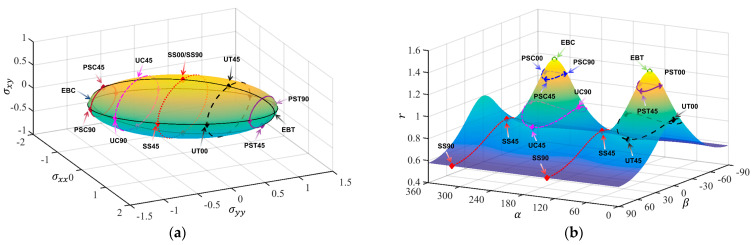
Three-dimensional von Mises yield surface under plane stress: (**a**) $\left(\sigma_{xx},\sigma_{yy},\sigma_{xy}\right)$ space, (**b**) $\left(\alpha ,\beta ,r\right)$ space.

Therefore, different stress states have corresponding values in $\left(\sigma_{xx},\sigma_{yy},\sigma_{xy}\right)$ space and $\left(\alpha ,\beta ,r\right)$ space along different rolling angles according to the above formula, and the yield surfaces in the two spaces can be converted into each other. The Mises yield surfaces in the two spaces are shown in Figure 2. The curves of different colors in Figure 2 represent stress states such as uniaxial tension (UT), uniaxial compression (UC), plane strain tension (PST), plane strain compression (PSC), shear (SS), equiaxial biaxial tension (EBT), and equiaxial biaxial compression (EBC). It can be found that the surfaces of Figure 2a,b are symmetrical about the $\sigma_{xy}=0$ plane and the β=0 plane, respectively, so half of the surface is studied to improve the calculation efficiency. In this study, α and β are selected as input variables and *r* is used as the output variable. Compared to the traditional method of using three stress components or stress invariants as input variables, it is more unique and monotonic.

### 2.2. The Calibration Results for Four Yield Functions

The anisotropic plastic behavior of cold rolled sheet metals is generally determined through uniaxial tensile tests conducted in different directions, as shown in Figure 1b. The anisotropy of cold rolled sheet metal is one of its most important properties. To model the plastic behavior that depends on direction, many anisotropic yield functions have been introduced. Modeling accuracy is a significant factor affecting the accuracy and reliability of numerical analyses of plastic deformation in sheet metal. This section introduces the macroscopic manifestation of anisotropic plastic behaviors of sheet metals, the typical anisotropic yield functions, and a brief summary of these yield functions. For most sheet metals with moderate anisotropy, uniaxial tensile tests are suggested to be conducted along three directions RD, DD, and TD. It needs to stretch the dogbone specimens along every 15° for strong anisotropic alloys, such as 2090 T3, 3104-H19, 2024-O aluminum alloy, etc. [31]. The anisotropic plastic behaviors of sheet metals are macroscopically represented by anisotropy in strength and plastic deformation. The anisotropy in plastic deformation is denoted by the *r*-value, while the anisotropy in strength is denoted by the yield stresses along different stretching directions, so the results of AA2090 T3 are shown in Table 1.

There are many anisotropic yield functions, which vary greatly in form and characteristics. In general, the selection of appropriate yield functions for specific metals and applications is crucial for the reliability of simulation results. The selection of a yield function is generally based on the target materials, application, experimental data available, etc. Before comparison and evaluation, the key features of some popular yield functions are summarized in Table 2 below. It is very difficult to evaluate all the yield functions and there are no widely accepted standards to evaluate the yield functions. Banabic [32,33] summarizes some important factors that must be taken into account when choosing a yield criterion, including prediction accuracy, numerical calculation efficiency, difficulty of calibration, and user-friendliness. However, these factors are sometimes mixed together, and the accuracy increases with the number of mechanical parameters required for the identification process. For example, high accuracy generally requires the yield function to be in a more complicated form with more anisotropic parameters, more experimental data with high experimental costs for parameter calibration, reduced efficiency in numerical analysis, etc. In general, accuracy is the most important consideration when choosing a yield function. Therefore, four typical anisotropic yield functions are selected to calibrate and verify the results of AA2090-T3.

The Hill48 and yld89 yield functions can describe the anisotropy of yield stress strength and *r*-values for RD, DD, TD, and equibiaxial tension, and the anisotropic parameters can be calculated analytically. The Hill48 yield function is the simplest anisotropic yield function, and its conjugate equivalent plastic strain can also be easily and explicitly calculated. It cannot properly model the strength around the plane strain due to the quadratic form. The anisotropic forms of the yld89 and Yld2000-2d yield functions that take into account the shear stress effect are used for the numerical simulation of shell elements. The Yld2004-18p yield function can simultaneously describe the anisotropy of strength and *r*-values of strongly anisotropic metals under uniaxial and equiaxial tension. The Yld2004-18p yield function can simultaneously describe the anisotropy of strength and *r*-values of anisotropic metals under uniaxial and equiaxial tension. For strongly anisotropic metals, the Yld2004-18p function can predict six or eight ears. It can be used for shell and solid elements under plane stress and spatial loading conditions. However, the experimental costs for anisotropic parameter calibration are high, and the computational costs are high due to the high complexity of the function.

**Figure 3:**
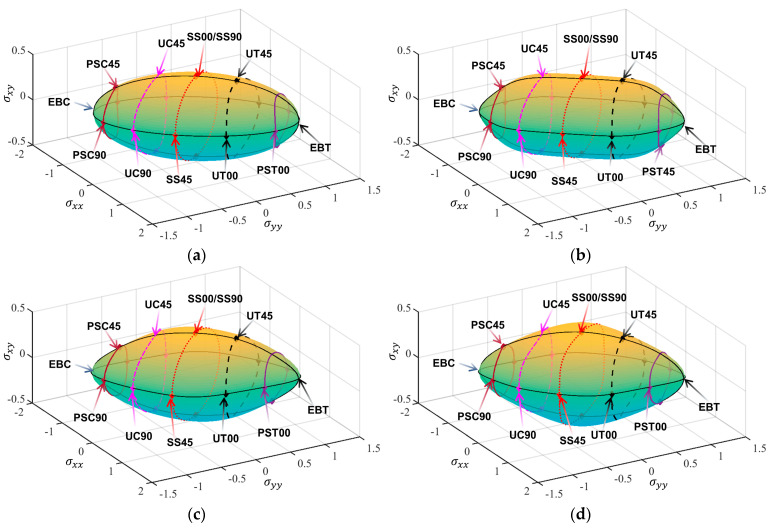
Three-dimensional yield surface under plane stress in $\left(\sigma_{xx},\sigma_{yy},\sigma_{xy}\right)$ space: (**a**) Hill48, (**b**) Yld89, (**c**) Yld2000-2d, (**d**) Yld2004-18p.

**Figure 4:**
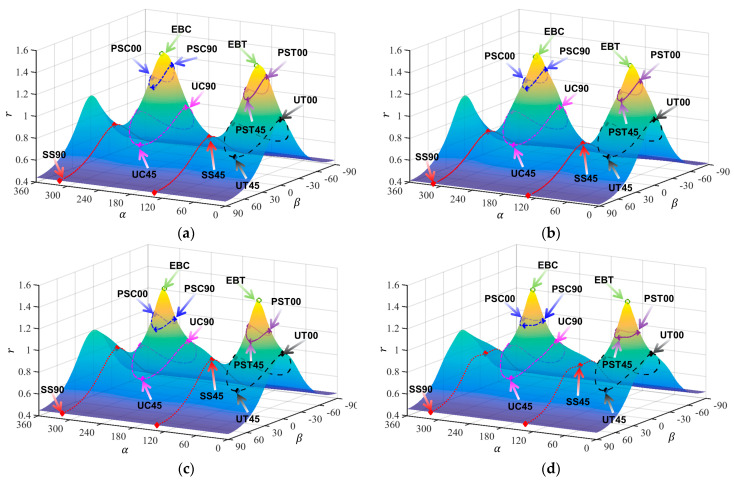
Three-dimensional yield surface under plane stress in $\left(\alpha ,\beta ,r\right)$ space: (**a**) Hill48, (**b**) Yld89, (**c**) Yld2000-2d, (**d**) Yld2004-18p.

Table 3, Table 4, Table 5 and Table 6 summarize the calibration parameters of the four yield functions for AA2090-T3. Figure 3 and Figure 4 show the shapes of the yield surfaces of the four yield functions in $\left(\sigma_{xx},\sigma_{yy},\sigma_{xy}\right)$ space and $\left(\alpha ,\beta ,r\right)$ space and the corresponding positions of different stress states. It can be seen from the results of Figure 3 that the results of Hill48 and Yld89 functions are relatively regular ellipses, while the yield surfaces of Yld2000-2d and Yld2004-18p functions reflect higher degrees of freedom. The results of Figure 4 show that the curve shapes of different yield functions under plane strain in the range of β∈(±13.26) are completely different.

**Figure 5:**
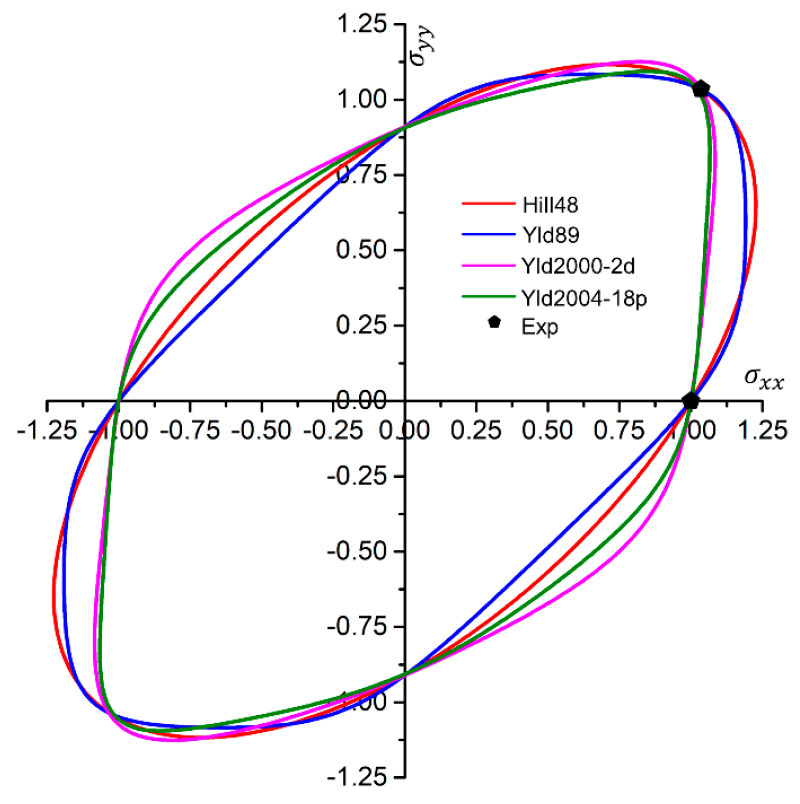
Comparison of yield loci of the four yield functions.

**Figure 6:**
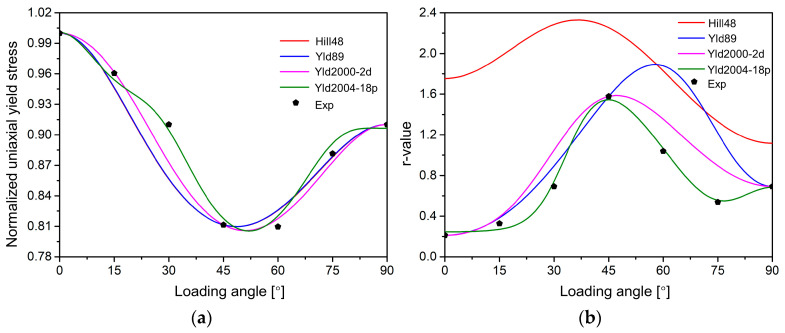
Comparison of the predicted results and experimental values of the four yield functions at different loading angles: (**a**) yield stress, (**b**) *r*-values.

Figure 5 compares the predicted yield surfaces of the four yield functions to the experimental data. From the comparison to experimental data points, all yield functions are able to describe the initial yield surface. The Yld2000-2d and Yld2004-18p functions can more flexibly capture the curvature of the yield surface of AA2090-T3 under the plane strain tension compared with the Hill48 and Yld89 yield functions. Figure 6 shows the comparison of the predicted uniaxial yield stress and *r*-value of the four yield models along different load directions. The calibration differences in the *r*-values of the four yield functions are relatively large compared to the yield stress. The yield stress calibrated by the Hill48 and Yld89 yield functions varies almost the same along different angles *θ*, but the *r*-values predicted by the two functions are significantly different. The predicted *r*-values of the Hill48 yield function are very different from the experimental results. The yield stress and *r*-values predicted by the Yld2004-18p model are significantly better in agreement with the experimental data. Therefore, the Yld2004-18p model with more parameters has the highest accuracy among the four models.

## 3. Machine Learning

### 3.1. Neural Network Model Architecture

According to the transformation of variables in the $\left(\sigma_{xx},\sigma_{yy},\sigma_{xy}\right)$ and $\left(\alpha ,\beta ,r\right)$ spaces in Section 2.1, the final modeling problem is rewritten as

(22) $$f:\left(\alpha ,\beta \right)\to r$$

Therefore, α and β are input variables and r is the output variable in the training of neural network. The traditional yield equation is calibrated based on the anisotropic stress and *r*-value, and about 4000 data points are obtained in the $\left(\alpha ,\beta ,r\right)$ space. Then, the prediction results of the yield surfaces of the two spaces $\left(\sigma_{xx},\sigma_{yy},\sigma_{xy}\right)$ and $\left(\alpha ,\beta ,r\right)$ based on the output variable r can be obtained. The convergence conditions were evaluated by the root mean square error (RMSE) during training. The quality and selection strategy of training data directly affect the performance and generalization ability of the neural network model. Variable transformation not only reduces the training difficulty of the neural network model but also makes the training results reliable and unique.

**Figure 7:**
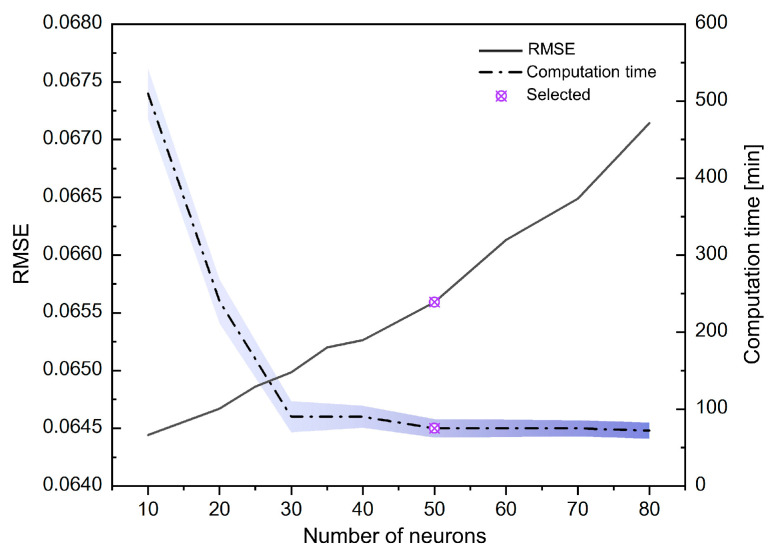
The calibration error and finite element calculation time of neural network model with different numbers of neurons.

According to the research of Shang et al. [16], the algorithm-optimized single-hidden-layer FCNN model has the advantage of taking into account high calibration accuracy and low finite element calculation time. In this study, analysis and comparison were performed on a common performance computer (Intel(R) Core(TM) i7−9700 CPU @ 3.00 GHz 3.00 GHz; 16.0 GB RAM; 4 CPU for all the simulations). According to the results in Figure 7, it can be seen that the finite element calculation time is positively correlated with the number of neurons. Purple indicates error bands at different numbers of neurons. However, the calibration accuracy has an obvious inflection point when the number is 30, and the calibration accuracy no longer improves significantly after the number is greater than 50. Finally, a single-layer FCNN model with 50 neurons optimized by particle swarm algorithm was selected. Since each yield equation generates approximately 3720 input data points, the training set is randomly divided into training data and test data at a ratio of 90–10% to ensure the predictive power of the model. In the same network structure, the parameters chosen have a great impact on the calibration results. The tansig activation function is used between the input and hidden layers, and the purelin activation function is used between the hidden and output layers. The epoch of the model is set to 3000 and the optimal combination of learning rate and momentum is adjusted to 0.01 and 0.9, which led to higher convergence speed and training stability.

**Figure 8:**
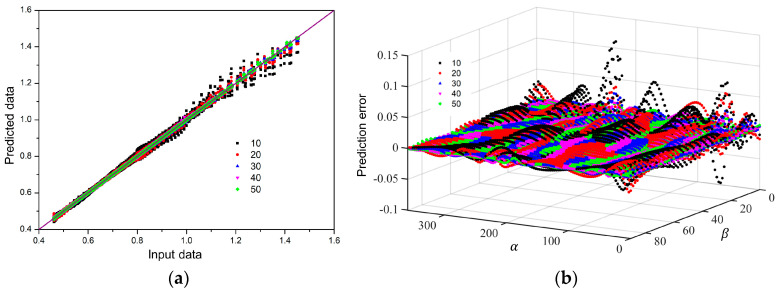
Analysis of the prediction results of the FCNN model for the Yld2004-18p yield function: (**a**) comparison of predicted data and input data, (**b**) prediction error with different numbers of neurons.

Figure 8 shows the comparison results of input data and predicted data with different numbers of neurons. It can be found that the data points gradually concentrate near the diagonal line as the number of neurons increases. Additionally, almost all of the green data points are distributed on a diagonal line at the number 50. At the same time, the results of Figure 8b also show that the prediction error gradually decreases as the number of neurons increases. The error fluctuation of β near 0 is very large when the number of neurons is relatively small, and the green data points are a plane near 0 when the number is 50.

**Figure 9:**
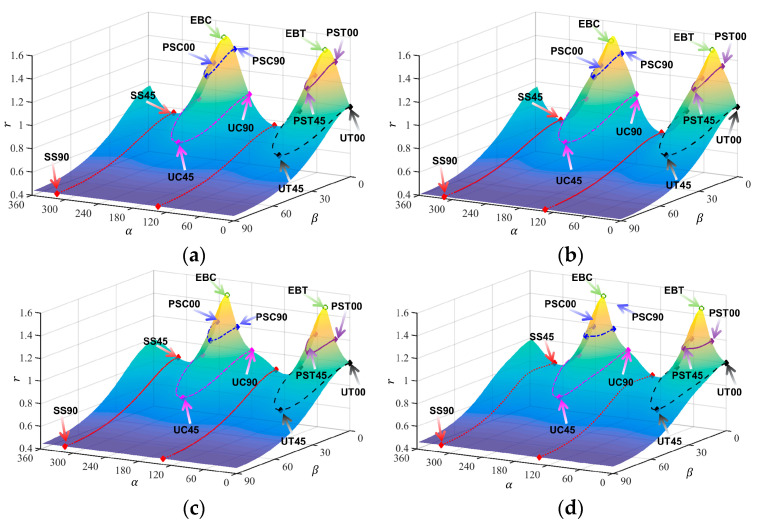
Three-dimensional yield surface predicted by the FCNN model under plane stress in (*α*, *β*, *r*) space: (**a**) Hill48, (**b**) Yld89, (**c**) Yld2000-2d, (**d**) Yld2004-18p.

**Figure 10:**
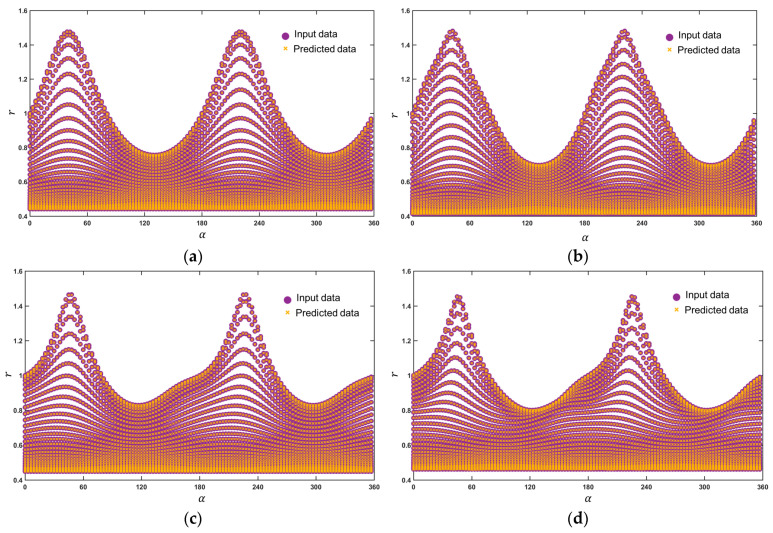
Comparison of input data and predicted data of FCNN model: (**a**) Hill48, (**b**) Yld89, (**c**) Yld2000-2d, (**d**) Yld2004-18p.

Figure 9 is the result of direct prediction by the FCNN model. It can be seen that the calibration data points of different models are all on the surface. Moreover, the smooth surface is similar to Figure 4, which further verifies the reliability of the model prediction. Figure 10 is a cross-sectional view of Figure 9, and the prediction errors of the input data and the predicted data are both within 0.01. It can be seen that the selected FCNN model strategy can well reproduce the characteristics of the input data, laying the foundation for subsequent calculations and conversions.

### 3.2. Analysis and Calculation of Prediction Results

**Figure 11:**
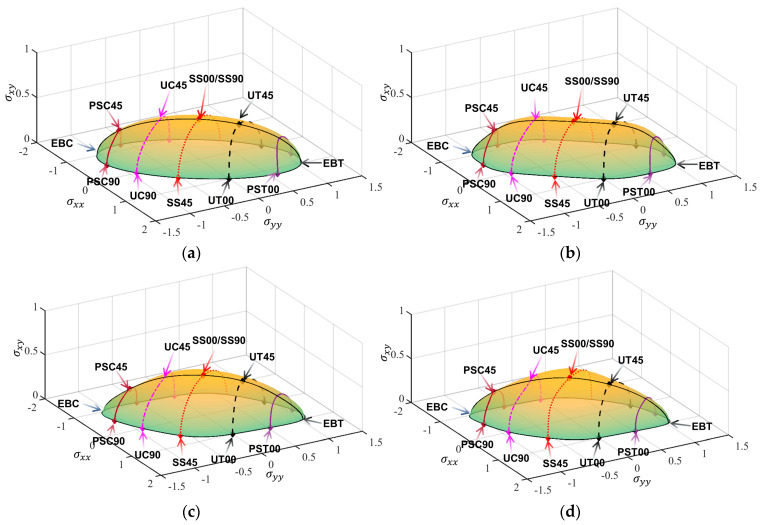
Three-dimensional yield surface predicted by the FCNN model under plane stress in $\left(\sigma_{xx},\sigma_{yy},\sigma_{xy}\right)$ space: (**a**) Hill48, (**b**) Yld89, (**c**) Yld2000-2d, (**d**) Yld2004-18p.

The accurate characterization of anisotropic yield behavior is of great significance in fields such as sheet metal forming. As an emerging phenomenological model, the neural network covers the anisotropy properties of different yield functions. In this way, all traditional models can be covered by a subroutine, making the application of finite elements more universal. According to the content in Section 2.1, the predicted results in the $\left(\alpha ,\beta ,r\right)$ space are transformed to obtain the surface of the $\left(\sigma_{xx},\sigma_{yy},\sigma_{xy}\right)$ space as shown in Figure 11.

The plastic deformation of metal sheets is very sensitive to the loading direction, so it exhibits anisotropy of strength and plastic deformation on a macro-scale. Equation (8) is normalized according to $\sigma_{\theta }=1$ to calculate $r_{0}$ as follows:

(23) $$\left\{\begin{array}{c} \sigma_{xx}^{0}={cos}^{2}\theta \\ \sigma_{yy}^{0}={sin}^{2}\theta \\ \sigma_{xy}^{0}=\sin\theta \cos\theta \end{array}\right.$$

(24) $$r_{0}=\sqrt{{\left(\sigma_{xx}^{0}\right)}^{2}+{\left(\sigma_{yy}^{0}\right)}^{2}+{\left(\sigma_{xy}^{0}\right)}^{2}}$$

**Figure 12:**
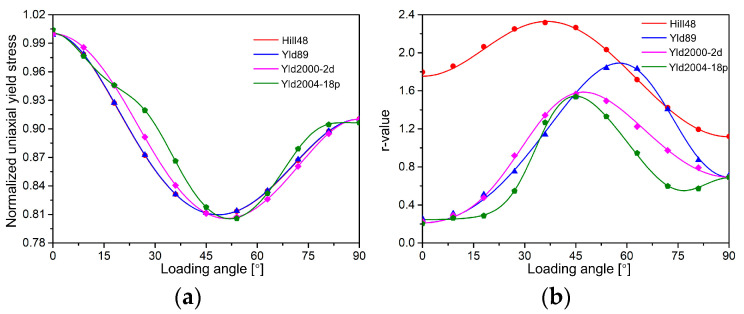
Comparison of the prediction results of the FCNN model and the calibration results of traditional yield functions at different loading angles: (**a**) yield stress, (**b**) *r*-values.

Therefore, the normalized yield stress of uniaxial tension calculated according to Equation (25) is $\sigma_{predict}$. The results of the four yield equations calculated in [0, *π*/2] are shown in Figure 12a. The results show that the solid line results predicted by FCNN are consistent with the scattered point results calculated by theory.

(25) $$\sigma_{predict}=\frac{r_{predict}}{r_{0}}$$

Then, the partial derivative of the yield function f is solved according to the definition, and the value of Δ in Equation (26) is taken as $10^{-5}$. The results of the *r*-value calculated according to Equation (17) are shown in Figure 12b. The predicted results of the *r*-value of the four yield functions are the same as the theoretical prediction results in Figure 6, which verifies the rationality and accuracy of the prediction of the FCNN model based on the traditional yield equation.

(26) $$\begin{array}{c} r_{∆xx}=\sqrt{{\left(\sigma_{xx}^{0}+∆\right)}^{2}+{\left(\sigma_{yy}^{0}\right)}^{2}+{\left(\sigma_{xy}^{0}\right)}^{2}}\\ r_{∆yy}=\sqrt{{\left(\sigma_{xx}^{0}\right)}^{2}+{\left(\sigma_{yy}^{0}+∆\right)}^{2}+{\left(\sigma_{xy}^{0}\right)}^{2}}\\ r_{∆xy}=\sqrt{{\left(\sigma_{xx}^{0}\right)}^{2}+{\left(\sigma_{yy}^{0}\right)}^{2}+{\left(\sigma_{xy}^{0}+∆\right)}^{2}} \end{array}⟹\begin{array}{c} \frac{\partial f}{{\partial \sigma }_{xx}}=\left(\frac{r_{predict}}{r_{∆xx}}-\frac{r_{predict}}{r_{0}}\right)/∆\\ \frac{\partial f}{{\partial \sigma }_{yy}}=\left(\frac{r_{predict}}{r_{∆yy}}-\frac{r_{predict}}{r_{0}}\right)/∆\\ \frac{\partial f}{{\partial \sigma }_{xy}}=\left(\frac{r_{predict}}{r_{∆xy}}-\frac{r_{predict}}{r_{0}}\right)/∆ \end{array}$$

## 4. Crystal Plasticity

In this section, crystal plasticity simulations of anisotropy and different stress states are used as inputs to the neural network model to predict the yield surface in $\left(\sigma_{xx},\sigma_{yy},\sigma_{xy}\right)$ space and $\left(\alpha ,\beta ,r\right)$ space. First, the crystalline plastic constitutive model considering slip is described, and the material parameters are the results of oxygen-free copper calibrated by Huang et al. [34]. Then, the training process is discussed, and the microscopic crystal plasticity and macroscopic yield equation are combined by the FCNN model.

It is believed that the dislocation slip is the main mechanism of polycrystalline copper deformation during plastic deformation, which means that crystal deformation includes elastic and plastic deformation. According to Zhu et al. [35], the multiplication decomposition of the deformation gradient tensor *F* is as follows:

(27) $$F=F^{e}F^{p}$$

where $F^{e}$ and $F^{p}$ represent the elastic and plastic deformation gradients, respectively.

The velocity gradient L can be decomposed into the sum of the elastic velocity gradient $L^{e}$ and the plastic velocity gradient $L^{p}$:

(28) $$L=\dot{F}\cdot F^{-1}={\dot{F}}^{e}\cdot F^{e^{-1}}+F^{e}\cdot \left({\dot{F}}^{p}\cdot F^{p^{-1}}\right)\cdot F^{e^{-1}}=L^{e}+L^{p}$$

The unit vectors $S^{\left(\alpha \right)}$ and $m^{\left(\alpha \right)}$, respectively, represent the slip direction and normal to slip plane of the *α*th slip system in the undeformed configuration. The calculation of the two vectors is as follows:

(29) $$s^{*\left(\alpha \right)}=F^{e}\cdot S^{\left(\alpha \right)}$$

(30) $$m^{*\left(\alpha \right)}=m^{\left(\alpha \right)}\cdot F^{e^{-1}}$$

The following is the plastic velocity gradient $L^{p}$ considering the slip mechanism:

(31) $$L^{p}=\sum_{\alpha =1}^{n}{\dot{\gamma }}^{\left(\alpha \right)}S^{*\left(\alpha \right)}m^{*\left(\alpha \right)}$$

where *n* is the total number of slip systems and ${\dot{\gamma }}^{\left(\alpha \right)}$ is the plastic shear strain rate caused by slip.

The power law model is used to characterize the strain hardening of crystalline materials based on Schmid’s law, which is expressed as

(32) $${\dot{\gamma }}^{\left(\alpha \right)}={\dot{\gamma }}_{0}^{\alpha }{\left|\frac{\tau^{\alpha }}{g^{\alpha }}\right|}^{\frac{1}{m}}sign\left(\tau^{\alpha }\right)$$

where $\tau^{\alpha }$ is the shear stress, *m* is the rate sensitivity index, and $g^{\alpha }$ is an internal variable describing the strength of the current slip system.

The deformation strengthening effect in the model is usually expressed in the following incremental form:

(33) $${\dot{g}}^{\alpha }=\sum h_{\alpha \beta }\left|{\dot{\gamma }}^{\beta }\right|$$

where $h_{\alpha \beta }$ is the hardening modulus of slip.

The hardening modulus reflects the increase in slip resistance caused by the proliferation and interaction of dislocations during deformation [36].

(34) $$\left\{\begin{array}{c} h_{\alpha \beta }=\left[q+\left(1-q\right)\delta_{\alpha \beta }\right]h_{\beta }\\ h_{\beta }=h_{0}{sech}^{2}\left|\frac{h_{0}\gamma }{\tau_{s}-\tau_{0}}\right|\\ \gamma =\sum_{\alpha }\int_{0}^{t}\left|{\dot{\gamma }}^{\alpha }\right|dt \end{array}\right.$$

where $h_{0}$ is the initial hardening modulus, γ is the accumulated shear strain of the slip system, $\tau_{0}$ is the yield stress, and $\tau_{s}$ is the shear stress caused by the large plastic flow.

(35) $$C_{11}=\frac{E\left(1-2v^{2}\right)}{\left(1-3v^{2}-2v^{3}\right)}$$

(36) $$C_{12}=\frac{E\left(v+v^{2}\right)}{\left(1-3v^{2}-2v^{3}\right)}$$

(37) $$C_{44}=G$$

The $C_{11}$, $C_{12}$, $C_{44}$ are calculated according to Equations (35)–(37). The crystal plasticity constitutive model based on the dislocation density is implemented in the commercial finite element software (Abaqus 6.14). The material parameters of the crystal plasticity model for polycrystalline copper are listed in Table 7, following the works of Huang [36] and Liu et al. [37].

**Figure 13:**
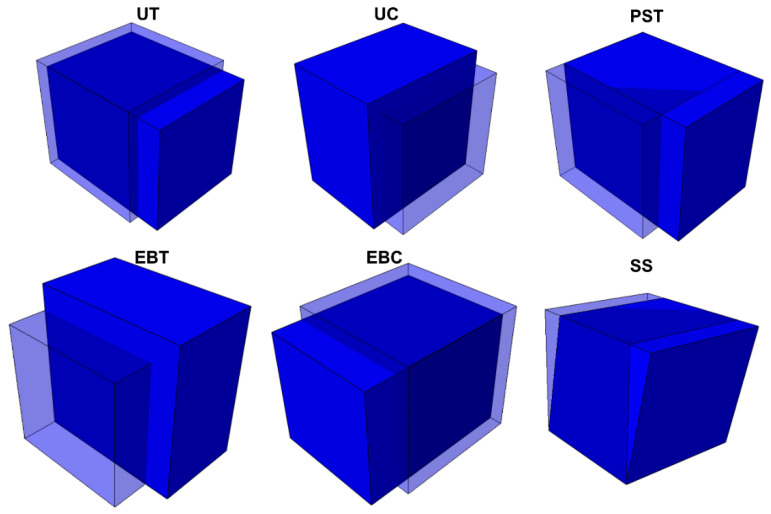
Simulation of crystal plasticity under different stress states.

**Figure 14:**
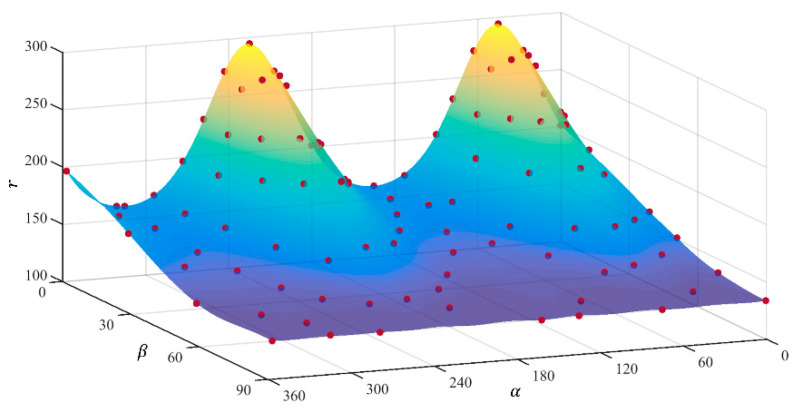
The 3D yield surface predicted by the FCNN model based on the simulation results of crystal plasticity is in $\left(\alpha ,\beta ,r\right)$ space.

**Figure 15:**
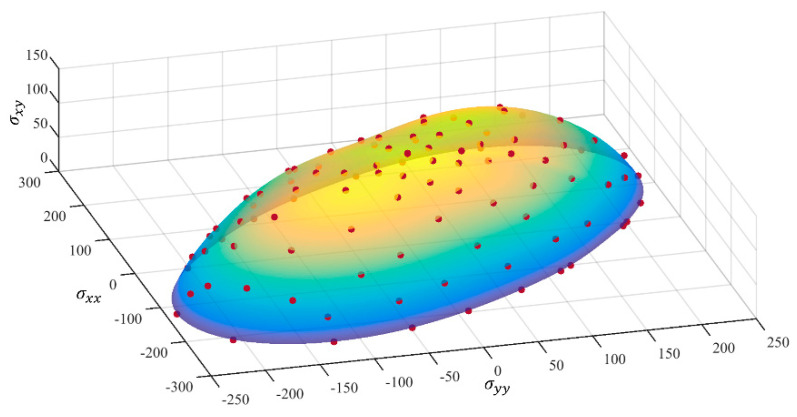
The 3D yield surface predicted by the FCNN model based on the simulation results of crystal plasticity is in $\left(\sigma_{xx},\sigma_{yy},\sigma_{xy}\right)$ space.

Figure 13 shows the crystal plasticity simulation of different stress states based on the material parameters in Table 7. In the macroscopic simulation, the results of the traditional model calibration are used as the input of the FCNN model, and then only one FCNN-based subroutine can cover all traditional models. In the microscopic field, the yield stress of different stress states and loading directions obtained by CPFEM simulation is directly used as the input of the FCNN model and then the results in Figure 14 and Figure 15 are obtained. It can be found that the fitting results of the yield model based on FCNN can reflect the characteristics of the input data with a high degree of freedom.

For the implementation framework, the results of macro-constitutive model calibration or crystal plasticity simulation are first used as the input of the neural network model, and the experimental data are calibrated by calling the MATLAB toolbox (neural net fitting) to determine the parameters of the FCNN model. The structure and parameters of the network are adjusted by the optimization algorithm so that the prediction error converges to the expected value. The calibrated FCNN model is embedded in ABAQUS/Explicit, and then the calibrated weights and thresholds are input into VUMAT written in Fortran. In short, the training function of the neural network is realized through MATLAB calibration, and the prediction function of the neural network is realized through the finite element subroutine.

Crystal plasticity simulations predict plastic deformation behavior under different loading conditions based on the microstructural characteristics of crystals (such as lattice type, slip system, texture, etc.), which help to understand the nature of metal deformation from a microscopic scale. By comparing the simulation results to the experimental data, the rationality and reliability of the crystal plasticity model that introduces dislocation density evolution information are further verified. The simulation of crystal plasticity of polycrystalline materials needs to consider complex phenomena, such as texture evolution and non-uniform deformation, which increase the complexity and uncertainty of the simulation. Therefore, it is necessary to preprocess the simulation results. The distribution shape of the traditional model was analyzed in $\left(\sigma_{xx},\sigma_{yy},\sigma_{xy}\right)$ space and $\left(\alpha ,\beta ,r\right)$ space, and the fitting results of high-order polynomial equations were used as the input of the ANN model to ensure the reliability of the predicted results.

## 5. Conclusions

The anisotropic behavior of AA2090-T3 is calibrated by four yield equations, and the yield surface characteristics are studied in $\left(\sigma_{xx},\sigma_{yy},\sigma_{xy}\right)$ and $\left(\alpha ,\beta ,r\right)$ spaces. The mapping relationships between α,β and r are established, and the feasibility of the FCNN model as a macroscopic anisotropic yield function is verified. Further, different stress states and anisotropic yield stresses are simulated by crystal plasticity as an input of the FCNN model, and the anisotropic behavior at the microscopic scale is further calibrated. The conclusions are summarized as follows:

1. The mutual conversion of anisotropic yield function in $\left(\sigma_{xx},\sigma_{yy},\sigma_{xy}\right)$ and $\left(\alpha ,\beta ,r\right)$ spaces is realized through theoretical calculation, and the corresponding positions of different stress states and loading directions are pointed out.
2. Higher quality training data are obtained through variable conversion, and the mapping relationships between α,β, and r are constructed. The yield stress and *r*-values are calculated according to the prediction results of the FCNN model.
3. The FCNN model combined with the traditional anisotropic yield functions characterizes the macroscopic anisotropic behavior, and the characteristics of different yield functions are covered based on a neural network subroutine.
4. Data points of different stress states and loading directions are generated through the crystal plasticity simulation, and the corresponding yield surface is obtained by the FCNN model. The FCNN model is directly regarded as a yield function, which can show higher flexibility, and the FCNN model can achieve the unification of macro- and micro-models.

## Figures and Tables

**Table 1:** Normalized yield stress and *r*-values for AA2090-T3.

$\sigma_{0}/\sigma_{0}$	$\sigma_{15}/\sigma_{0}$	$\sigma_{30}/\sigma_{0}$	$\sigma_{45}/\sigma_{0}$	$\sigma_{60}/\sigma_{0}$	$\sigma_{75}/\sigma_{0}$	$\sigma_{90}/\sigma_{0}$	$\sigma_{b}/\sigma_{0}$
1.0000	0.9605	0.9102	0.8114	0.8096	0.8815	0.9102	1.0350
$r_{0}$	$r_{15}$	$r_{30}$	$r_{45}$	$r_{60}$	$r_{75}$	$r_{90}$	$r_{b}$
0.2115	0.3269	0.6923	1.5769	1.0385	0.5384	0.6923	0.67

**Table 2:** Key features of popular anisotropic yield functions.

Yield Function	# of Anisotropic Parameters ^①^	Precisely Modeled Experimental Data Under Plane Stress	Curvature Control for BCC/FCC	Stress Spaces	Form ^②^
Hill48	6@3d,4@2d	*σ*_0_, *σ*_45_, *σ*_90_, *σ*_*b*_ or *σ*_0_, *r*_0_, *r*_45_, *r*_90_	no	3d	quadratic
Yld89	4@2d	*σ*_0_, *σ*_45_, *σ*_90_, *σ*_*b*_ or *σ*_0_, *r*_0_, *r*_45_, *r*_90_	yes	2d	③
Yld2000-2d	8@2d	*σ*_0_, *σ*_45_, *σ*_90_, *σ*_*b*_, *r*_0_, *r*_45_, *r*_90_, *r*_*b*_	yes	2d	PS
Yld2004-18p	18@3d,	*σ*_0_, *σ*_15_, *σ*_30_, *σ*_45_, *σ*_60_, *σ*_75_, *σ*_90_,	yes	3d	PS
	14@2d	*σ*_*b*_, *r*_0_, *r*_15_, *r*_30_, *r*_45_, *r*_60_, *r*_75_, *r*_90_, *r_b_*			

^①^ # of anisotropic parameters is based on associated flow rules. For non-associated flow rules with an identical potential, # of anisotropic parameters is almost doubled. ^②^ All the yield functions in the table are non-quadratic except Hill48. PS is short for principal stresses, which indicates that the yield function is in th eform of principal stresses or principal deviatoric stresses. SI is short for stress invariants, which means that the yield function is in a form of the two deviatoric stress invariants *J*_2_ and *J*_3_, or the two stress invariants of linearly transformed stress tensors. ③ The Yld89 and BCC2000 functions are neither in a form of PS nor SI. Readers are suggested to go back to the corresponding sections for details of these two yield functions.

**Table 3:** Anisotropic coefficients of Hill48 for AA2090-T3.

*F*	*G*	*H*	*N*
1.1405	0.7264	1.2735	5.1421

**Table 4:** Anisotropic coefficients of Yld89 for AA2090-T3.

*h*	*c*	*m*	*p*
1.0986	1.5136	0.4863	1.2512

**Table 5:** Anisotropic coefficients of Yld2000-2d for AA2090-T3.

$\beta_{1}$	$\beta_{2}$	$\beta_{3}$	$\beta_{4}$	$\beta_{5}$	$\beta_{6}$	$\beta_{7}$	$\beta_{8}$
0.1249	1.6807	0.8656	0.9729	1.0518	0.8602	1.2356	1.4333

**Table 6:** Anisotropic coefficients of Yld2004-18p for AA2090-T3.

$c_{12}^{\prime }$	$c_{13}^{\prime }$	$c_{21}^{\prime }$	$c_{23}^{\prime }$	$c_{31}^{\prime }$	$c_{32}^{\prime }$	$c_{44}^{\prime }$	$c_{55}^{\prime }$	$c_{66}^{\prime }$
−0.0698	0.9364	0.0791	1.0030	0.5247	1.3631	1.0237	1.0690	0.9543
$c_{12}^{\prime \prime }$	$c_{13}^{\prime \prime }$	$c_{21}^{\prime \prime }$	$c_{23}^{\prime \prime }$	$c_{31}^{\prime \prime }$	$c_{32}^{\prime \prime }$	$c_{44}^{\prime \prime }$	$c_{55}^{\prime \prime }$	$c_{66}^{\prime \prime }$
0.9811	0.4767	0.5753	0.8668	1.1450	−0.0792	1.0516	1.1471	1.4046

**Table 7:** Material parameters used in the simulations.

$C_{11}$ [GPa]	$C_{12}$ [GPa]	$C_{44}$ [GPa]	$h_{0}$ [MPa]	$g_{s}$ [MPa]	$g_{0}$ [MPa]	*q*	${\dot{\gamma }}_{0}\left[s^{-1}\right]$	*m*
168.4	121.4	75.4	541.59	109.5	60.8	1	0.001	0.1

## Data Availability

The data that support the findings of this study are available from the corresponding authors upon reasonable request.
